# Influences of the G2350A polymorphism in the ACE Gene on cardiac structure and function of ball game players

**DOI:** 10.1186/1477-5751-11-6

**Published:** 2012-01-12

**Authors:** Yongwoo Jang, Sung Min Kim

**Affiliations:** 1 College of Physical Education, Hanyang University, Seoul 133-791, Korea; 2 College of Pharmacy, Seoul National University, Seoul 151-742, Korea

**Keywords:** ACE, Ball Game Players, Echocardiography

## Abstract

**Background:**

Except for the I/D polymorphism in the angiotensin I-converting enzyme (ACE) gene, there were few reports about the relationship between other genetic polymorphisms in this gene and the changes in cardiac structure and function of athletes. Thus, we investigated whether the G2350A polymorphism in the *ACE *gene is associated with the changes in cardiac structure and function of ball game players. Total 85 healthy ball game players were recruited in this study, and they were composed of 35 controls and 50 ball game players, respectively. Cardiac structure and function were measured by 2-D echocardiography, and the G2350A polymorphism in the *ACE *gene analyzed by the SNaPshot method.

**Results:**

There were significant differences in left ventricular mass index (LVmassI) value among each sporting discipline studied. Especially in the athletes of basketball disciplines, indicated the highest LVmassI value than those of other sporting disciplines studied (p < 0.05). However, there were no significant association between any echocardiographic data and the G2350A polymorphism in the *ACE *gene in the both controls and ball game players.

**Conclusions:**

Our data suggests that the G2350A polymorphism in the *ACE *gene may not significantly contribute to the changes in cardiac structure and function of ball game players, although sporting disciplines of ball game players may influence the changes in LVmassI value of these athletes. Further studies using a larger sample size and other genetic markers in the *ACE *gene will be needed.

## Background

It is known that athletes who take regular and intensive physical exercise experience change in the cardiac structure and function, which is called an athlete's heart [1], as a part of physiological adaptation to physical exercise. It shows basically different symptoms from the abnormal condition of cardiac structure and function caused by complication of cardiovascular diseases such as essential hypertension. That is to say, as to cardiac structure, while it shows change such as increase in the thickness of ventricular wall caused by increase in the volume of ventricle and hypertrophy of myocardium, as to cardiac function, it is known to show increase in stroke volume and decrease in resting heart rate [2].

Such physiologically adaptive aspect which occurs to athletes, however, is known to depend on period, intensity and type of physical exercise and time. In case of athletes who do aerobic exercises such as marathon or long distance running repeatedly, an aspect of increase in the left ventricular internal dimension at end-diastole or increase in stroke volume is shown. In case of athletes who take muscle strengthening exercise such as weight lifting or wrestling, it was known that not only the thickness of left ventricular posterior wall and interventricular septum increase but also the left ventricular mass increases [3].

But, in case of most of sports, it is more likely for athletes to take composite exercises of two types in combination rather than to do simple aerobic exercise or muscle strengthening exercise separately and it is especially so in case of ball game players. According to the results of studies performed up to now, though it is known that athletes who take composite exercises show increase in left ventricular mass, in cardiac structure, and low resting heart rate as well as reduction in cardiac output, in cardiac function, as both types of exercises have influence, it is required to study effects of composite exercise on cardiac structure and function by analyzing more fractionized exercise items as objects because it shows different aspects depending on the ratio and contents of types of exercises taken.

It is also known that even the athletes who take exercises under similar program with similar intensity for a similar period show inter-individual difference in the change of cardiac structure and function, of which the cause is known to be at least partly attributable to genetic factors [4].

As it is known that renin-angiotensin system not only performs an important role in cardiovascular function through regulation of blood pressure and fluid homeostasis but also regulates growth of myocardial cells by expressing components of the system in cardiac cells, the genes encoding components of such physiological systems are attracting public attention as the potential candidate genes which affect cardiac structure and function [5]. Among the components which consist this system, as angiotensin I converting enzyme (ACE) is known to not only convert angiotensin (I), which is a precursor to angiotensin II by being expressed in diverse body tissues including heart tissue, but also inactivate bradykinin to eventually promote growth of cardiac cells, the biggest number of studies have been performed in relation to change in cardiac structure and function such as left ventricular hypertrophy caused by complication of cardiovascular diseases such as essential hypertension as well as left ventricular hypertrophy shown for athlete groups [6].

It is known that *ACE *gene which encodes ACE is located at human chromosome 17 and there exists many polymorphisms including a polymorphism formed by insertion/deletion of 287 bp existing in intron 16 of this gene [7,8]. Though the biggest number of studies have been performed for I/D polymorphism among various kinds of polymorphisms existing in *ACE *gene in relation to the change in cardiac structure and function including various cardiovascular diseases and left ventricular hypertrophy (LVH) up to now as it shows significant association with ACE level in tissues and serum, the results of studies have shown difference between study groups [9-11].

But, though there are a number of other polymorphisms in *ACE *gene than I/D polymorphism and further study is required for clinical association of these polymorphisms, the situation is that not as many studies as I/D polymorphism have been performed up to now [8,12].

Zhu *et al*., (2001) have performed association study of 13 kinds of polymorphisms existing in *ACE *gene with serum ACE level and blood pressure using them as genetic markers on 1,343 subjects of 332 Nigerian families. As a result of the study, they presented that G2350A polymorphism existing in exon 17 of this gene had the most powerful influence on serum ACE level more than that of I/D polymorphism. This result means that the influence of I/D polymorphism on serum ACE level may not be direct genetic effect but may be the result of linkage disequilibrium with an allele [8]. Accordingly, it is required to develop new genetic markers which can exercise more powerful genetic effect and G2350A polymorphism has risen as one of new alternative genetic markers which can satisfy such expectation. Recently, result of several studies on the relation of G2350A polymorphism with cardiovascular diseases, such as essential hypertension and myocardial infarction as well as with left ventricular hypertrophy, have been reported [13-15].

But, the situation is that, in relation to the influence of *ACE *gene on cardiac structure and function shown for athlete groups, while the majority of study is of those of I/D polymorphism as subject [4,6,16-20], there is almost no study on other kinds of polymorphism of this gene including G2350A polymorphism.

For this reason, we selected ball game players who are taking composite exercises such as soccer, baseball, basketball, volleyball and ice hockey as subjects of this study and attempted to analyze whether G2350A polymorphism existing in *ACE *gene has significant effect on change of cardiac structure and function shown for athletes engaged in such game items.

## Methods

### Study subjects

The participants of this study are consisted of 83 subjects, 50 ball game players (soccer 16, baseball 10, basketball 8, volleyball 8 and ice hockey 8) who have exercise carrier at least 6 years and 33 controls that do not have any career of physical exercise. All these participants are of late teens or early 20s with good health, whose clinical characteristics are shown in Table 1. Written informed consent was obtained from all subjects, and this study was also approved by institutional review board.

**Table 1:** Physical characteristics of study subjects

Variables	Controls(n = 33)	Athletes(n = 50)
Age(year)	22.3 ± 2.0	21.3 ± 1.2*
Height(cm)	181.3 ± 4.8	182.0 ± 8.0
Weight(kg)	74.1 ± 9.2	76.3 ± 8.0
BMI(kg/m^2^)	22.5 ± 2.2	23.0 ± 1.8

*Abbreviation: *BMI = body mass index. *p < 0.05.

### Measurements of cardiac structure and function

**Figure 1:**
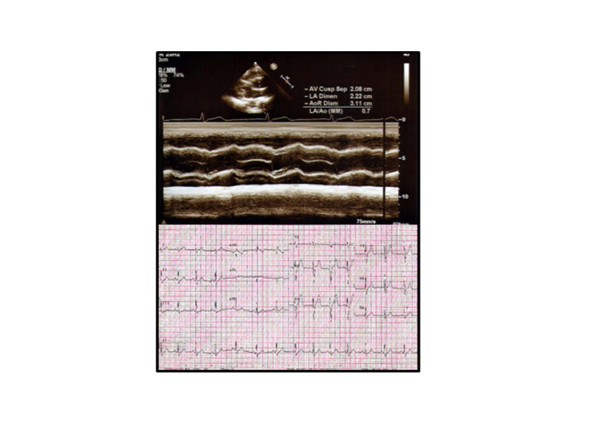
**An echocardiogram of a subject**.

The anthropological parameters of the participants of this study were measured using IN-BODY 3.0 (Bio-Space, Co. Ltd., Korea) and the characteristics related to cardiac structure and function such as aortic root (AR), left ventricular internal dimension at end-diastole (LVIDd), left ventricular internal dimension at end-systole (LVIDs), left ventricular mass (LVmass), left ventricular mass index (LVmassI), stroke volume (SV), resting heart rate (HR_rest_), cardiac output (CO) and percent of fractional shortening (FS [%]) were measured using M-mode echocardiography in accordance with the instruction established by American Society of Echocardiography (ASE) (Figure 1). Also, if the LVmassI value of the subject is 125 g/m^2 ^or more, the subject was judged to have left ventricular hypertrophy [21].

### Genetic analysis

In order to analyze G2350A polymorphism existing in *ACE *gene, about 3~5 ml of venous blood is taken from peripheral vein of each subject who participated in this study, which was then moved to a blood-collecting tube containing EDTA, an anticoagulant, and stored in refrigerator until genetic analysis was performed.

**Figure 2:**
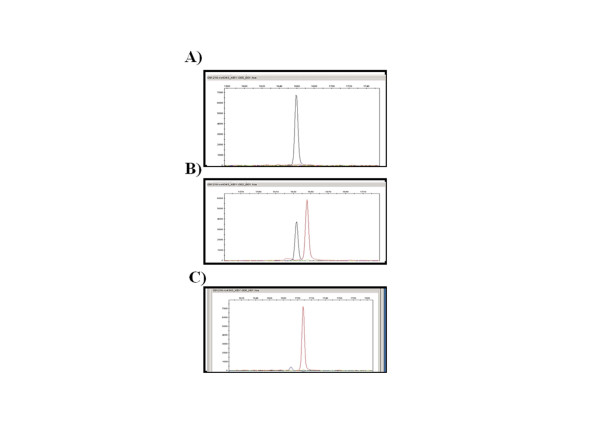
**Detection of G2350A polymorphism in the *ACE *gene by SNaPshot method**. A) GG genotype, B) GA genotype and C) AA genotype.

Isolation of total genomic DNA from venous blood of subjects was performed using Miniban Automatic Blood DNA Isolation Kit (Bionex, Co. Ltd., Korea), which is an automated device, and analysis of G2350A polymorphism (rs#4343) existing in *ACE1 *gene was performed in SNaPshot method using total genomic DNA of the subjects isolated in above method (Figure 2).

### Biochemical assay

After extracting the blood collected from peripheral veins of subjects to serum isolation tube, serum lipid and glucose levels were measured from the serum isolated. In case of serum total cholesterol (TC), triglyceride and HDL-cholesterol, concentration was measured using an automated device, ADVIA 1650 autoanalyzer, together with enzymatic treatment and colorimetry and the concentration of serum LDL-cholesterol was estimated using the equation proposed by Friedewald, et al. 1972 [22]. Concentration of plasma glucose was measured using Hitachi 7180 autoanalyzer together with enzymatic treatment and colorimetry similarly with measurement of serum lipid concentration.

### Data analysis

Whether observed genotype of G2350A polymorphism of *ACE1 *gene is in Hardy-Weinberg equilibrium was analyzed by χ^2^-test together with the difference in frequency of genotype and allele consisting G2350A polymorphism of *ACE1 *gene between the control group and the ball game players or between players of each item among athlete groups.

Also, whether the 3 genotypes consisting G2350A polymorphism of *ACE1 *gene have association with echocardiographic data and serum biochemical parameters was analyzed by a one-way ANOVA test. The statistical significance level was set as α = 0.05 in this study and all statistical analysis was performed using SPSSWIN version 17.0 program.

## Results

### Prevalence of LVH according to sporting disciplines

The result of comparing the frequency of left ventricular hypertrophy (LVH) between ball game players, though basketball players showed the highest left ventricular mass index (LVmassI) and the highest frequency of LVH, no statistically significant difference was observed between each item (Table 2). But, all the ball game players who participated in this study showed higher LVmassI and higher frequency of LVH than the control group. In addition, in the result of comparing all the ball game players and the control group, athletes showed statistically higher mean value of LVmassI than controls (t = -2.175, p = 0.033).

**Table 2:** Left ventricular mass index in our subjects

Disciplines	**No**.	LVmassI	No. of LVH(%)
Soccer	16	117.3 ± 15.8	6(37.5)
Baseball	8	117.8 ± 15.4	3(37.5)
Basketball	10	126.6 ± 15.7	5(50.0)
Volleyball	8	117.4 ± 17.7	2(25.0)
Icehocky	8	121.1 ± 16.6	2(25.0)
Athletes	50	119.9 ± 15.9	18(36.0)
Controls	33	109.5 ± 27.6	5(15.2)
Total	83	115.7 ± 21.8	23(27.7)

There were significant difference in the values of left ventricular mass index between controls and ball game players (t = -2.175, p = 0.033).

### Distribution of the G2350A polymorphism in the ACE gene

Table 3 shows the difference between the result of investigating distribution of genotype and allele which consists G2350A polymorphism of *ACE1 *gene of control group and ball game player group. Though the result of investigating whether this polymorphism is in Hardy-Weinberg equilibrium showed that control group (χ^2 ^= 0.2650, df = 1, p = 0.6064), ball game player group (χ^2 ^= 0.0090, df = 1, p = 0.9251) and all the subjects (χ^2 ^= 0.0560, df = 1, p = 0.8122) are all in Hardy-Weinberg equilibrium, no statistically significant difference was observed between genotypes (χ^2 ^= 1.4375, df = 2, p = 0.4874) and allele frequency (χ^2 ^= 0.8555, df = 1, p = 0.3550) of two groups. Also, in the result of examining whether the genotypes consisting this genetic polymorphism (χ^2 ^= 0.2952, df = 2, p = 0.8628) and allele (χ^2 ^= 0.1089, df = 1, p = 0.7414) show any significant associations with LVH, neither the genotype nor allele showed any significant association with LVH (Table 4). Table 5 is the result of investigating distribution aspect of frequency of genotype and allele consisting G2350A polymorphism of *ACE *gene by each exercise item among ball game player group. The result showed that there is no statistically significant difference in genotypes (χ^2 ^= 7.0594, df = 10, p = 0.7198) and allele frequency (χ^2 ^= 2.9359, df = 5, p = 0.7099) of this genetic polymorphism between game items.

**Table 3:** Distribution of the G2350A polymorphism in the *ACE *gene in controls and ball game players

Genotype	Subject No.(%)	
	
	Controls(n = 33)	Athletes(n = 50)
GG	6(18.2)	7(14.0)
GA	18(54.5)	23(46.0)
AA	9(27.3)	20(40.0)
Total	33(100.0)	50(100.0)
	Χ^2 ^= 1.4375, df = 2, p = 0.4874	

**Allele**	**Chromosome No.(%)**	
	
	**Control(2n = 66)**	**Athletes(2n = 100)**

G	30(45.5)	37(37.0)
A	36(54.5)	63(63.0)
Total	66(100.0)	100(100.0)
	Χ^2 ^= 0.8555, df = 1, p = 0.3550	

**Table 4:** Distribution of the G2350A polymorphism in the *ACE *gene by LVH status

Genotype	Subject No.(%)	
	
	Normal(n = 60)	LVH(n = 23)
GG	9(15.0)	4(17.4)
GA	29(48.3)	12(52.2)
AA	22(36.7)	7(30.4)
Total	60(100.0)	23(100.0)
	Χ^2 ^= 0.2952, df = 2, p = 0.8628	

**Allele**	**Chromosome No.(%)**	
	
	**Normal(2n = 120)**	**LVH(2n = 46)**

G	47(39.2)	20(43.5)
A	73(60.8)	26(56.5)
Total	120(100.0)	46(100.0)
	Χ^2 ^= 0.1089, df = 1, p = 0.7414	

**Table 5:** Distribution of the G2350A polymorphism in the *ACE *gene in ball game players and controls

Disciplines	**No**.	Genotype No.(%)			Allele No.(%)	
	
		GG	GA	AA	G	A
Soccer	16	4(25.0)	6(37.5)	6(37.5)	14(43.8)	18(56.2)
Baseball	8	0(0.0)	5(62.5)	3(37.5)	5(31.3)	11(68.7)
Basketball	10	1(10.0)	4(40.0)	5(50.0)	6(30.0)	14(70.0)
Volleyball	8	2(25.0)	3(37.5)	3(37.5)	7(43.8)	9(56.2)
Icehocky	8	0(0.0)	5(62.5)	3(37.5)	5(31.3)	11(68.7)
Athletes	50	7(14.0)	23(46.0)	20(40.0)	37(37.0)	63(63.0)
Controls	35	6(18.2)	18(54.5)	9(27.3)	30(45.5)	36(54.5)
Total	85	13(15.7)	41(49.4)	29(34.9)	67(40.4)	99(59.6)
Χ^2^		7.0594			2.9359	
df		10			5	
p-value		0.7198			0.7099	

### Clinical influences of G2350A polymorphisms in the ACE gene

The result of investigating whether the 3 genotypes (GG, GA and AA) consisting G2350A polymorphism of *ACE *gene have significant associations with echocardiographic data and serum biochemical parameters in the control group (Table 6) and ball game player group (Table 7) Both groups showed no statistically significant association with echocardiographic data and serum biochemical parameters.

**Table 6:** Influences of the G2350A polymorphism in the *ACE *gene on cardiovascular structures and functions in controls

Variables	GG(n = 6)	GA(n = 18)	AA(n = 9)
Age(year)	21.5 ± 1.4	22.5 ± 2.0	22.4 ± 2.2
Height(cm)	182.3 ± 6.4	180.4 ± 4.0	182.2 ± 5.5
Weight(kg)	75.8 ± 15.7	73.7 ± 7.6	73.6 ± 7.8
BMI(kg/m^2^)	22.7 ± 3.6	22.6 ± 2.0	22.1 ± 1.6
AR(cm)	2.8 ± 0.2	2.9 ± 0.2	3.0 ± 0.1
LVIDd(cm)	5.1 ± 0.4	5.2 ± 0.3	5.2 ± 0.3
LVIDs(cm)	3.1 ± 0.4	3.3 ± 0.3	3.2 ± 0.2
LVmass(g)	243.7 ± 143.1	209.6 ± 41.2	203.0 ± 28.6
LVmassI	119.6 ± 55.4	108.5 ± 20.3	104.6 ± 12.0
SV(ml)	85.5 ± 17.4	87.4 ± 13.6	90.9 ± 13.0
HR_rest_(beat/min)	61.8 ± 10.3	67.7 ± 11.2	66.8 ± 14.3
CO(ml/min)	5271.5 ± 1400.8	5890.6 ± 1186.2	6068.9 ± 1516.9
FS(%)	39.0 ± 3.8	37.1 ± 4.2	38.6 ± 1.8
TC(mg/dl)	139.5 ± 20.0	171.5 ± 73.8	147.0 ± 19.8
TG(mg/dl)	87.5 ± 25.3	167.2 ± 233.0	62.4 ± 30.7
LDL-C(mg/dl)	78.5 ± 20.7	92.3 ± 49.0	85.8 ± 15.2
HDL-C(mg/dl)	43.5 ± 7.1	45.7 ± 7.5	48.7 ± 11.5
Glucose(mg/dl)	87.7 ± 8.4	85.1 ± 9.3	83.2 ± 15.2

*Abbreviations: *BMI = body mass index, AR = aortic root, LVIDd = left ventricular internal dimension at end-diastole, LVIDs = left ventricular internal dimension at end-systole, LVmass = left ventricular mass, LVmassI = left ventricular mass index, SV = stroke volume, HR_rest _= resting heart rate, CO = cardiac output, FS = percent of fractional shortening, TC = total cholesterol, TG = triglyceride, LDL-C = low density lipoprotein cholesterol, HDL-C = high density lipoprotein cholesterol.

**Table 7:** Influences of the G2350A polymorphism in the *ACE1 *gene on cardiovascular structures and functions in ball game players

Variables	GG(n = 7)	GA(n = 23)	AA(n = 20)
Age(year)	21.3 ± 1.4	21.2 ± 1.2	21.4 ± 1.1
Height(cm)	184.4 ± 5.2	179.6 ± 8.5	183.8 ± 7.8
Weight(kg)	77.0 ± 3.5	76.4 ± 9.0	75.9 ± 8.2
BMI(kg/m^2^)	22.7 ± 1.1	23.7 ± 2.1	22.4 ± 1.4
AR(cm)	2.9 ± 0.1	3.0 ± 0.3	3.1 ± 0.3
LVIDd(cm)	5.7 ± 0.3	5.4 ± 0.4	5.6 ± 0.3
LVIDs(cm)	3.7 ± 0.2	3.5 ± 0.4	3.5 ± 0.3
LVmass(g)	247.0 ± 35.6	230.6 ± 43.5	240.5 ± 37.4
LVmassI	124.0 ± 19.8	117.6 ± 16.8	121.0 ± 13.7
SV(ml)	113.6 ± 25.9	106.3 ± 25.3	112.0 ± 21.6
HR_rest_(beat/min)	56.6 ± 5.6	59.6 ± 9.9	56.6 ± 7.5
CO(ml/min)	6531.3 ± 2100.9	6303.9 ± 1725.5	6346.9 ± 1567.9
FS(%)	35.3 ± 2.1	35.9 ± 3.7	36.6 ± 3.1
TC(mg/dl)	141.6 ± 24.5	157.0 ± 26.5	160.6 ± 20.2
TG(mg/dl)	97.1 ± 38.1	105.5 ± 55.1	86.6 ± 39.7
LDL-C(mg/dl)	74.6 ± 18.0	86.8 ± 18.9	87.5 ± 17.5
HDL-C(mg/dl)	47.6 ± 10.6	49.2 ± 9.9	55.8 ± 11.0
Glucose(mg/dl)	90.6 ± 17.4	87.0 ± 11.5	87.1 ± 13.0

*Abbreviations: *BMI = body mass index, AR = aortic root, LVIDd = left ventricular internal dimension at end-diastole, LVIDs = left ventricular internal dimension at end-systole, LVmass = left ventricular mass, LVmassI = left ventricular mass index, SV = stroke volume, HR_rest _= resting heart rate, CO = cardiac output, FS = percent of fractional shortening, TC = total cholesterol, TG = triglyceride, LDL-C = low density lipoprotein cholesterol, HDL-C = high density lipoprotein cholesterol.

## Discussion

The objective of this study is to investigate G2350A polymorphism distribution of the *ACE *gene of ball game players as well as to analyze the effect of genetic polymorphism on the cardiovascular function of these subjects.

The result of investigating left ventricular mass index (LVmassI) and frequency of left ventricular hypertrophy (LVH) of ball game players such as soccer, baseball, basketball, volleyball and ice hockey, all the ball game players studied showed higher LVmassI value and higher LVH in average than those of the control group. This can be interpreted as result of change in cardiac structure and function caused by adaptation to intensive exercise taken by ball game players for a long period of time [23]. Also, although no statistical significance has been observed, the basketball players and ice hockey players showed higher LVmassI value than players of other ball games, but basketball players showed the highest frequency of LVH as well. This result is because of different exercise methods between ball games which causes the cardiac structure and function to show different adaptive aspects. That is to say, it seems that the players of ball games such as basketball and ice hockey where fierce body fighting and body contact take place show larger change in adaptive aspect of cardiac structure and function to physical exercise than players of other ball games.

As *ACE *gene has long been attracting public attention as a genetic predisposition which affects cardiac structure and function such as LVH, many genetic epidemiological studies have been performed, and especially studies to investigate association of I/D polymorphism existing in this gene with cardiac structure and function have been globally performed with diverse groups including athlete groups as subjects. In most of these studies executed with patients of cardiovascular disease as subjects, as well as athletes who mainly take aerobic exercise and athletes who mainly take resistant exercise, it was reported that all showed significant association with change in cardiac structure and function caused by physical exercise [4,6,16-20,24,25].

However, although I/D polymorphism of *ACE *gene is associated with genetic linkage, it has been well unknown actual genetic effect [7]. Therefore, we believe it is required to develop other new genetic markers which can exercise actual effect in this gene. Moreover, as various single nucleotide polymorphisms (SNP) in *ACE *gene are found to have more powerful effect of regulating serum ACE level than the I/D polymorphism, it will be very interesting to perform a study to analyze clinical association using G2350A polymorphism of *ACE *gene as the genetic marker [8].

Up to now, the first study result which analyzed clinical influence of G2350A polymorphism of *ACE *gene was the study performed by Mshmood *et al*., (2003), which analyzed association of G2350A polymorphism with essential hypertension with United Arab Emirates (UAE) population as subjects, where they reported that this genetic polymorphism showed significant association with essential hypertension [13]. Also, though Iqbal *et al*., (2004) analyzed whether this genetic polymorphism showed significant association with myocardial infarction with Pakistan population as subjects, they could not detect any significant association [14]. In relation to cardiac structure and function, Saeed *et al*., (2005) also analyzed association of this genetic polymorphism with left ventricular mass (LVH) using UAE population as subjects. As a result they reported significant association of this genetic polymorphism with LVH [10]. Furthermore, in the study performed by Pan *et al*., (2007) with Chinese population as subjects, we suggested that G2350A polymorphism of *ACE *gene may affect change in cardiac structure and function shown for athlete group. It showed significant association with LVH appearing as a complication caused by essential hypertension, though this genetic polymorphism did not show any significant association with essential hypertension [15].

Nevertheless, to our knowledge, as there is no particular result of study on the influence of G2350A polymorphism on change in cardiac structure and function appearing to athlete group because there has not been much genetic epidemiological study on G2350A polymorphism of *ACE *gene in comparison to I/D polymorphism, we analyzed what influence G2350A polymorphism of *ACE1 *gene has on the change in cardiac structure and function of ball game players.

The result of comparing frequency of genotypes and allele consisting G2350A polymorphism of *ACE *gene of the control group and ball game players participating in this study showed that the genotype frequency observed was in Hardy-Weinberg equilibrium.

However, when frequency of genotypes and allele of G2350A polymorphism of this gene of the control group and ball game players participating in this study was compared, the result showed no significant difference in frequency of two groups and of each ball game players. Also, in this study, analysis was performed to examine what influence G2350A polymorphism of *ACE *gene has on cardiovascular risk factors such as echocardiographic data and serum biochemical parameters of the groups. No statistically significant association has been observed in both groups. There are some possibilities and limitations that G2350A polymorphism of *ACE *gene was not correlated with athletic performance ability in this study.

One possibility may be interpreted as the difference among ethnic groups. When frequency of A alleles which consist G2350A polymorphism of *ACE1 *gene was compared with frequency of A alleles of other healthy ethnic groups studied up to now, the frequency of A alleles of the participants of this study was 0.60 showing higher value than those of UAE population (0.37~0.38) of Pakistan population (0.32) and Chinese population (0.43) [10, 13~15]. However, our study with Korean population as subjects did not show any significant genetic association with cardiac structure and function of ball game players. Therefore, we think that further study is required to be performed with other ethnic groups as subjects.

Other limitations could be caused by the number of participants and diversity of athletics. In this study, we composed of 35 controls and 50 ball game player. More participants may be required for this subject to get statistical significance. We also recruited single athletic players in this study. We need to perform to check whether genetic polymorphism of this gene has any significant association with cardiac structure and function of athletes who take aerobic exercise such as long distance running or marathon and athletes who take resistant exercises such as wrestling or weight lifting. Actually, the I/D polymorphism of *ACE *gene is associated with endurance athletics such as marathon runner, longer distance swimmer [26,27]. Therefore, we think that further study on athletes of various sporting disciplines is also required.

## Conclusions

In conclusion, our data suggest that the G2350A polymorphism in the *ACE *gene may not be a useful genetic marker on cardiac structure and function of ball game players. Further studies using larger sample size and other candidate genes will be required.

## Competing interests

The authors declare that they have no competing interests.

## Authors' contributions

YJ performed the statistical analysis, drafted and revised the manuscript. SK participated in the design of the study, coordination, and final approval of the article. All authors read and approved the final manuscript.
